# Supplementary material for: Detection of Human Papillomavirus DNA in Paired Peripheral Blood and Cervix Samples in Patients with Cervical Lesions and Healthy Individuals
Source: J Clin Med. 2021 Nov 8;10(21):5209. doi: 10.3390/jcm10215209 (PMC8584517; doi:10.3390/jcm10215209)
Supplement: Supplementary file 1 [file jcm-10-05209-s001.zip › jcm-1372137-supplementary.pdf]

Supplementary Table S1. Title.

Patient	Age	Residence	Ethnicity	Age at the first sexual relation	Number of pregancies	Number of sexual partners	Contraceptive use	Smoking	Previous STIs	HPV DNA in the blood	HPV DNA in the cervix	Healthy_CINI_CINII_CINIII
5	1	0	2	0	1	0	0	0	0	0	0	1
11	0	1	2	0	1	0	1	0	1	1	0	1
16	2	1	2	0	1	0	0	0	1	1	1	1
17	0	1	2	0	1	1	1	0	1	0	0	1
18	1	0	2	0		1	0	1	1	0	0	1
22	1	1	2	0	1	0	0	0	0	1	1	1
23	1	1	0	0	1	0	0	0	0	0	1	1
29	0	1	0	0	1	0	0	0	1	0	0	1
39	0	1	1	0		2	0	0	1	0	1	1
43	0	0	0	0	2	1	0	0	1	0	0	1
44	0	1	2	0	2	1	0	0	0	1	0	1
46	1	1	2	0	2	0	0	2	0	1	1	1
52	0	1	1	0		2	0	1	0	1	1	1
66	0	1	1	0	0	1	1	1		1	0	1
69	0	0	0	0	0	0	1	2	1	1	1	1
72	0		1	0	1	0	0	0		0	0	1
73	0	1	1	0		0	0	0		1	1	1
105	0	1	1	0		1	0	1	0	0	0	1
106	1	1	1	0	2	1	0	1	0	0	1	1
109	1	1	2	1	1	1	0	0	0	1	0	1
110	0	1	1	0	2	2	0	0	1	1	1	1
111	0	1	1	0	2	2	0	2	0	0	1	1
116	0	1	0	0		2	0	0	0	0	1	1
121	0	1	0	1	1	1	0	1	0	0	0	1
125	1	1	0	0	2	0	0	1	0	1	1	1
128	1	1	2	0	2	1	0	0	0	0	0	1
133	0	1	2	0	2	0	0	0	0	1	1	1
135	0	1	2	0	0	2	0	0	0	0	0	1
136	0	1	2	0	0	1	0	0	0	0	0	1
137	0	1	1	0	1	0	0	0	0	1	1	1

139	1	0	1	0	1	1	0	0	1	0	0	1
145	0	0	1	0	1	0	0	0	1	1	1	1
146	1	1	2	1	1	0	0	0	0	0	0	1
149	0	1	1	0	0		1	0	0	0	0	1
151	1	0	2	0	0	1	0	2	0	0	0	1
27	0	1	2	0	1	0	0	1	1	1	1	1
30	1	1	1	0	2	1	0	0	1	1	1	1
45	0	1	1	1		0	1	0	1	0	1	1
47	0	1	1	0		0	1	0	0	1	1	1
50	0	0	2	0	0	0	0	2	1	1	0	1
54	0	1	0	0		1	1	0	0	0	0	1
71	0		2	0	0	0	0	0	1	0	1	1
80	0	1	0	0	1	1	1	2		0	0	1
13	0	1	2	0	1	0	1	0	0	1	1	2
28	0	1	2	1	1	0	1	0	0	1	1	2
32	1	1	2	3		0	0	0	0	0	0	2
33	0	0	1	0	0	1	0	0	0	1	1	2
56	0	1	2	0		1	0	0	1	1	0	2
65	0	1	2	0		1	1	0	1	0	1	2
67	1	0	2	0	2	0	0	0		1	1	2
76	0	1	0	1	1	0	0	0	1	1	1	2
77	0	1	1	0	0	0	0	0		0	1	2
83	0		1	0	1	0	0	0		0	0	2
100	0	1	2	0	0	1	0	0	0	0	0	2
102	0	1	1	0	1		0	1	0	0	1	2
130	0	1	0	0		1	0	0	0	1	0	2
147	1	1	0	0	1	0	0	0	0	0	0	2
9	0	1	1	0	2	1	0	1	0	1	1	2
148	0	1	2	0		1	0	0	0	1	0	2
6	0	1	1	0	0	0	1	0	0	1	1	3
10	1	1	1	0	2	1	0	0	1	1	0	3
36	1	1	2	0	1	2	0	0	0	1	1	3
61	1	1	1	0	2		0	0		1	1	3
70	1	1	2	1		1	0	0		1	1	3
88	0		1	2			0	0		0	1	3
90	1	1	2	0	1	1	0	0	0	1	0	3

92	1	1	0	0	2	0	0	0	0	1	0	3
93	0	1	1	0	1	2	1	0	0	0	0	3
3	0	0	1	0		0	0	0	0	0	0	0
4	0	1	2	0	1	0	0	0	0	0	0	0
8	1	1	1	1	2	0	0	0	0	1	0	0
12	0	1	1	0		1	1	0	0	1	0	0
14	0	1	0	1		1	0	0	0	1	1	0
21	0	1	2	0	1	0	0	0	0	0	0	0
24	0	1	1	2	1	0	0	0	0	1	0	0
25	0	1	2	0	0	1	1	0	0	0	0	0
31	1	1	2	1	1	0	0	0	0	0	0	0
35	0	1	1	0	1	0	0	0	0	1	1	0
51	1	1	1	0	2	0	0	0	0	1	0	0
53	0	1	0	1	1	0	0	0	0	1	0	0
58	1	1	1	0		1	0	0	0	1	1	0
59	0	1	1	0		0	1	0	0	0	0	0
60	1	1	2	1	0	1	0	0	0	0	0	0
63	2	1	1	0	2	0	0	0		1	0	0
64	0	0	1	0	1	0	0	0		1	1	0
75	0	1	1	0		1	0	0		1	1	0
81	0	1	1	0	1	0	0	0		0	0	0
82	0	1	2	1		0	1	0		0	0	0
85	0	1	1	0	1	2	1	1		0	0	0
86	0	1	1	0	1	1	0	0		1	0	0
91	0	1	1	0		2	0	0	0	1	0	0
95	1	1	2	0	1		0	0	0	1	0	0
96	1	1	2	0			0	0	0	0	0	0
98	1	1	0	2	1	0	0	0	0	0	0	0
99	1	1	0	0		0	0	0	0	0	0	0
104	0	1	1	0	0	1	0	1	0	1	0	0
107	0	1	1	0	0	0	0	0	0	0	0	0
108	2	1	0	0	2	0	0	0	0	0	0	0
112	0	1	1	0	1	2	0	0	0	0	0	0
113	0	1	1	0	1	0	1	0	0	0	0	0
114	1	1	2	1	1	0	0	0	0	0	0	0
115	1	1	2	0	1	0	0	2	1	1	1	0

118	1	1	2	0	1	1	0	2	0	0	0	0
119	0	1	1	0	0	2	0	0	0	0	0	0
120	0	1	0	0	0	0	0	0	0	1	0	0
122	0	1	2	0		1	1	0	0	1	1	0
123	0	1	2	0	1	1	0	1	0	0	0	0
126	1	1	0	0	1	0	0	0	0	1	1	0
127	0	1	1	0	1	1	0	1	0	0	0	0
129	1	1	1	0	1	1	0	2	0	0	0	0
131	1	1	2	0		1	0	0	0	1	1	0
132	1	1	1	0		1	0	0	0	1	1	0
134	1	1	0	1	1	1	0	1	0	0	0	0
141	1	1	1	1	2	0	0	0	0	0	0	0
142	1	1	1	1		0	0	0	0	0	0	0
143	0	1	2	1	0	2	0	0	0	0	0	0
150	1	0	1	1	1	0	0	2	0	0	0	0
152	1	1	0	0	0	1	0	0	0	1	1	0
153	1	1	2	0	1	0	0	0	0	1	1	0
156	1	1	2	1		0	0	0	0	1	1	0
157	1	1	2	0	1	2	0	2	0	1	0	0
158	0	1	1	0	0	1	1	0	0	1	0	0
159	0	0	2	1	1	1	0	0	0	0	0	0
160	1	0	2	0	2	0	0	0	0	0	0	0
161	0	1	0	0		1	0	0	0	0	0	0
162	0	0	2	0	0	0	0	0	0	0	0	0
163	0	1	1	1	1	0	1	0	0	0	0	0
168	0	0	2	0	1		0	0	0	1	0	0
169	0	1	2	0	1		0	0	0	0	0	0
170	0	1	1	0	0		1	0	0	1	0	0
171	0	1	1	0		2	1	0	0	0	0	0
20	1	0	2	0	1	0	0	0	0	0	0	0
49	0	1	2	1	0	0	1	0	0	1	1	0
62	1	1	2	0		0	0	0		1	1	0
68	0	1	2	0	1	0	0	0		1	0	0
74	0	1	1	0	0	1	0	0		1	1	0
87	1	1	1	0	1	0	0	1		0	0	0
94	1	1	2	0	2	1	0	0	0	1	1	0

97	1	1	1	1	1	2	0	0	0	0	0	0
				LEGEND								
Patient	Age	Residence	Ethnicity	Age at he first sexual relation	Number of pregancies	Number of sexual partners	Contraceptive use	Smoking	Previous STIs	HPV DNA in the blood	HPV DNA in the cervix	Healthy_CINI_CINII_CINIII
Number	0 - <45	0 - Rural	0 - Black	0-<20	0-1	0-1	0-No	0-No	0-No	0-No	0-No	0-Without lesions
	1- 40-60	1 - Urban	1 - Other	1-20-30	1 - 2 or 3	1 - 2 or 3	1 -Yes	1-Yes	1-Yes	1-Yes	1-Yes	1-CIN I
	2- >60		2 - White	2-30-40	2->4	2->4						2-CIN II
				3->40								3-CIN III

Supplementary Table S2. Title.

Patient	Viral load in the cervix	Viral load in the blood	Physical status in the blood	Physical status in the cervix	Healthy_CINI_CIN II_CIN III
58	14	34.89	Mixed	Mixed	Healthy
62	8.3	8.52	Episomal	Mixed	Healthy
63	0.12	8	Integrated	Mixed	Healthy
64	78	46.47	Integrated	Integrated	Healthy
74	99.7	2.76	Episomal	Mixed	Healthy
75	35.3	17.24	Mixed	Integrated	Healthy
115	0.12	33	Integrated	Mixed	Healthy
122	3.6	479.6	Integrated	Integrated	Healthy
125	0.14	10.37	Episomal	Mixed	Healthy
131	16.1	5.75	Mixed	Integrated	Healthy
132	6.1	60.35	Mixed	Integrated	Healthy
152	15.5	16.03	Mixed	Episomal	Healthy
153	163.1	10.6	Episomal	Episomal	Healthy
156	10.6	10.21	Mixed	Episomal	Healthy
158	4	83.89	Mixed	Episomal	Healthy
170	246.5	9.92	Episomal	Mixed	Healthy
172	0.68	3.47	Episomal	Episomal	Healthy
6	138.00	17.12	Mixed	Mixed	CIN III
9	68.10	4.38	Mixed	Integrated	CIN III
16	0.19	4	Episomal	Mixed	CIN III
30	24.30	3.65	Episomal	Integrated	CIN II
47	18.90	3.91	Mixed	Mixed	CIN I
52	15.10	21.58	Episomal	Mixed	CIN I
61	42.20	4.21	Mixed	Episomal	CIN III
67	3.90	4.09	Mixed	Integrated	CIN II
69	1.00	5.05	Mixed	Episomal	CIN I
92	7.13	11.69	Episomal	Mixed	CIN III
136	0.15	9.87	Mixed	Episomal	CIN I
137	0.20	7.31	Mixed	Integrated	CIN I
